# Much More Beyond Motor Symptoms – The Social Symptoms of Parkinson’s Disease

**DOI:** 10.1192/j.eurpsy.2025.1799

**Published:** 2025-08-26

**Authors:** M. Andrade, M. Magalhães

**Affiliations:** 1 Hospital Júlio de Matos - Unidade Local de Saúde São José, Lisbon, Portugal

## Abstract

**Introduction:**

Parkinson’s disease (PD) is the second most common neuropsychiatric disorder, affecting approximately 6.1 million people worldwide. Its incidence has increased over the last two decades, with a higher rate observed in industrialized countries, suggesting that pollution and social isolation may contribute. Some studies even identify loneliness as an independent risk factor. PD is characterized by a slow progression of disability, often lasting decades, with significant impact on both individuals and caregivers. In addition to its hallmark motor symptoms (rigidity, bradykinesia, and tremor), neuropsychiatric symptoms are now better understood, and some researchers have also identified social symptoms.

**Objectives:**

Our aim is to describe the social symptoms of PD and assess the impact of these symptoms on patients’ quality of life.

**Methods:**

A narrative literature review was conducted through bibliographic research in the PubMed and Google Scholar databases, using the terms “Parkinson’s disease,” “social symptoms,” and “social impact.”

**Results:**

The so-called social symptoms arise from various changes that occur in PD, affecting both the verbal and non-verbal components of communication. “Facial masking” results from facial muscle bradykinesia, leading to a diminished ability to produce facial expressions. Research has shown that individuals with PD have a reduced capacity to modulate emotional expressions, which are crucial for eliciting empathy and facilitating social interactions. Additionally, patients demonstrate difficulties in recognizing emotions, possibly due to a dysfunctional theory of mind (Embodied Simulation Theory). Reduced prosody leads to monotonous, emotionless speech, which contributes to negative self-perception and a feeling of impaired communication abilities. Furthermore, in more advanced stages of PD, there appears to be a deficit in interpreting prosody.

These factors contribute to stigma, dehumanization, and social isolation among PD patients, with significant consequences for their quality of life, which has been demonstrated in several studies.

**Conclusions:**

Social symptoms, though often overlooked, are prevalent in PD and substantially reduce patients’ quality of life. It is crucial to further investigate these symptoms and develop interventions to mitigate their impact.

**Disclosure of Interest:**

None Declared

